# First Evidence of Intraclonal Genetic Exchange in Trypanosomatids Using Two *Leishmania infantum* Fluorescent Transgenic Clones

**DOI:** 10.1371/journal.pntd.0003075

**Published:** 2014-09-04

**Authors:** Estefanía Calvo-Álvarez, Raquel Álvarez-Velilla, Maribel Jiménez, Ricardo Molina, Yolanda Pérez-Pertejo, Rafael Balaña-Fouce, Rosa M. Reguera

**Affiliations:** 1 Departamento de Ciencias Biomédicas, Universidad de León, Campus de Vegazana, León, Spain; 2 Unidad de Entomología Médica, Servicio de Parasitología, Centro Nacional de Microbiología, Instituto de Salud Carlos III, Majadahonda, Madrid, Spain; Lancaster University, United Kingdom

## Abstract

**Background:**

The mode of reproduction in *Leishmania* spp has been argued to be essentially clonal. However, recent data (genetic analysis of populations and co-infections in sand flies) have proposed the existence of a non-obligate sexual cycle in the extracellular stage of the parasite within the sand fly vector. In this article we propose the existence of intraclonal genetic exchange in the natural vector of *Leishmania infantum*.

**Methodology/Principal findings:**

We have developed transgenic *L. infantum* lines expressing drug resistance markers linked to green and red fluorescent reporters. We hypothesized whether those cells with identical genotype can recognize each other and mate. Both types of markers were successfully exchanged within the sand fly midgut of the natural vector *Phlebotomus perniciosus* when individuals from these species were fed with a mixture of parental clones. Using the yellow phenotype and drug resistance markers, we provide evidence for genetic exchange in *L. infantum*. The hybrid progeny appeared to be triploid based on DNA content analysis. The hybrid clone analyzed was stable throughout the complete parasite life cycle. The progress of infections by the hybrid clone in BALB/c mice caused a reduction in parasite loads in both spleen and liver, and provided weight values similar to those obtained with uninfected mice. Spleen arginase activity was also significantly reduced relative to parental strains.

**Conclusions/Significance:**

A *L. infantum* hybrid lineage was obtained from intraclonal genetic exchange within the midgut of the natural vector, suggesting the ability of this parasite to recognize the same genotype and mate. The yellow hybrid progeny is stable throughout the whole parasite life cycle but with a slower virulence, which correlates well with the lower arginase activity detected both *in vitro* and *in vivo* infections.

## Author Summary

Leishmaniasis is one of the most important human neglected parasitic diseases worldwide. When it appears in the visceral form it has the most aggressive outcome, and is fatal if left untreated. The current mode of reproduction of these parasites is under discussion, from propagation to a sexual reproduction process. Here, we describe for the first time the intraclonal genetic exchange between two transgenic *L. infantum* fluorescent strains in their natural vector *Phlebotomus perniciosus*. Hybrid clones displayed parental phenotypic traits such as yellow fluorescence and double-drug resistance. The DNA content of the hybrid lineage was 3n, while the parental lineage was 2n. The hybrid clone was able to undergo all the morphogenic transitions required to complete a full life cycle to infect a susceptible mammalian host, and maintained both phenotype and genotype. However, its capacity to generate the same lesions as those observed with parental clones in BALB/c mice was reduced.

## Introduction

*Leishmania* is a unicellular digenetic parasite causative of several devastating zoonotic diseases in poor and developing countries. It can survive in diverse environments from the sand fly vector (promastigotes) to the mammalian host (amastigotes) where temperature, pH and other conditions are extremely different. In humans, once the disease is acquired several clinical forms can be manifested. Visceral leishmaniasis is the most aggressive and prevalent disease caused by *Leishmania donovani* in both Asia and Africa, whereas diseases caused by *L. infantum* are endemic to countries of the Mediterranean basin and Latin America. The disease manifests as organ swelling (specifically targeting liver, spleen and bone marrow) and may be deadly if left untreated [1].

For years, the reproductive mode of *Leishmania* has been assumed to be predominantly clonal, based on strong linkage disequilibrium (LD) [2], [3]. Genetic population studies on different human strains of *Leishmania (Viannia) braziliensis* revealed substantial heterozygote deficit, which is inconsistent with a strictly clonal model of reproduction.

Alternatively a clonal/sexual reproduction and possible inbreeding is proposed [4]. In the above-mentioned studies *Leishmania* was considered “diploid”, however aneuploidy is now proposed as the norm rather than the exception both for lab strains [5] and natural isolates [6] (for reviews see [7]–[8]). Nonetheless, LD analysis does not require the knowledge of ploidy [9]. These studies clash with other authors proposing sexual reproduction [10]–[12]. In a focus of cutaneous leishmaniasis in Turkey the number of meioses per mitosis was estimated to be similar to the frequency of mating in co-infected sand fly studies [6].

In parallel, genetic exchange was demonstrated in landmark experiments by Beverleys' group following co-infections of *Phlebotomus duboscqi* sand flies with two strains of *L. major*. However, attempts to repeat the crosses of the parental lines during *in vitro* co-culture or co-infection in BALB/c mice were unsuccessful [10]. A subsequent study by the same group using four *L. major* strains from across its geographic range within both natural (*P. duboscqi*) and unnatural (*Lutzomyia longipalpis*) sand fly vectors confirmed that genetic exchange has no inter-specific barriers [11]. Another co-infection study in sand flies using genetically modified *L. donovani* strains, allowed the visualization and recovery of the progeny after experimental crosses, but the hybrids could not be propagated for further genotyping studies [12].

In support of the experimental work, there are some examples of naturally occurring hybrid genotypes observed in field isolates that involve different species of the *L. (Viannia)* subgenus [13]. *L. (Viannia.) braziliensis/L. (V.) peruviana* hybrids have been identified by microsatellite typing [14] and *L. (V.) braziliensis/L. (V.) guyanensis* hybrids are not uncommon [15]–[17]. Natural hybrids involving other species such as *L. major* and *L. arabica* have been reported [18], [19]. *Leishmania* hybrids have also been identified from very distant species; *L. major* and *L. infantum* [20], and *L. donovani* and *L. aethiopica* [21].

Since mating events have been proved by genetic and experimental studies, our goal is to explore whether *Leishmania* is able to perform recombination between the same genotype during the mating process. We have chosen a *L. infantum* strain isolated from an infected dog in Spain, classified as MCAN/ES/1996/BCN150 zymodeme MON-1. The use of two drug-selectable markers linked to fluorescent reporters (red and green), is an approach that relies on the production of yellow fluorescent hybrids as an identifiable biomarker of mating between two individuals from the same strain.

## Methods

### Mice and parasites

All experimental animal procedures described in this manuscript were carried out in strict accordance with the Spanish (Ley 32/2007) and European Union Legislation (2010/63/UE). The used protocols were approved by the Animal Care Committee of the University of León (Spain).

Female BALB/c mice (6–8 weeks old) were sourced from Harlan Interfauna Iberica SA (Barcelona, Spain) and housed in specific-pathogen-free facilities for this study. *L. infantum* (strain MCAN/ES/96/BCN 150) promastigotes were obtained from J.M. Requena (Centro de Biología Molecular Severo Ochoa, Madrid, Spain). Parasites were routinely cultured at 26°C in M199 medium supplemented with 25 mM HEPES pH 6.9, 10 mM glutamine, 7.6 mM hemin, 0.1 mM adenosine, 0.01 mM folic acid, 1× RPMI 1640 vitamin mix (Sigma), 10% (v/v) heat-inactivated foetal calf serum (FCS) and antibiotic cocktail (50 U/ml penicillin, 50 µg/ml streptomycin).

### Generation of fluorescent-transgenic *Leishmania infantum*

Two fluorescent *L. infantum* strains were generated in order to overexpress two different fluorescent proteins; mCherry (λ exc. 587 nm; λ em. 610 nm), which was kindly provided by Dr Roger Y. Tsien – Departments of Pharmacology and Chemistry & Biochemistry, UCSD (USA) and Citrine (λ exc. 516 nm; λ em. 529 nm), which was kindly provided by Dr. Juan Llopis – Facultad de Medicina and Centro Regional de Investigaciones Biomédicas, University of Castilla-La Mancha (Spain). For the *CITRINE-HYGROMYCIN (CTN-HYG)* construct, the 720-bp *CTN* coding region was amplified by PCR using pcDNA3 (Invitrogen) as template and the oligonucleotides RBF613 and RBF614 as primers (Table 1 contains all primers used in this work). The amplified fragment was cut with *BglII-NotI* and inserted into the pLEXSY-hyg2 vector (Jena Bioscience GmbH, Germany) to yield pLEXSY-*CTN*-*HYG* construct.

**Table 1:** Oligonucleotides used in this work.

Oligo No.	Sequence^a^^,^ b	Purpose^c^
RBF613	ccgCTCGAGgaAGATCT **CCACC**ATGGTGAGCAAGGGCGAGG	Citrine F
RBF613	ataagaatGCGGCCGCTTACTTGTACAGCTCGTCCATG	Citrine R
RBF634	ccgCTCGAGgaAGATCT **CCACC**ATGGTGAGCAAGGGCG	mCherry F
RBF600	ataagaatGCGGCCGCTTACTTGTACAGCTCGTCCATGC	mCherry R
RBF630	CTTGTTTCAAGGACTTAGCCATG	5′integration F
RBF644	CTCGTGTGAGCGTTCGCG	3′integration F
RBF645	CTACCTTGTTACGACTTTTGC	3′integration R
RBF676	gcTCTAGA **CCACC**ATGGCCAAGCCTTTGTCTCAAGAA	BSD F
RBF677	ggACTAGTTTAGCCCTCCCACACATAACCAG	BSD R
RBF772	ataagaatGCGGCCGCCCTCCTCCTCCTTTCTTGTTCC	utr2 F
RBF773	gcTCTAGAGGCTGCTGTGGAGGTGTGTAG	utr2 R

a Underlined sequence indicates restriction site.

b Bold sequence indicates optimized translation initiation sequence.

c Orientation: F, forward; R, reverse.

For the *mCHERRY-BLASTICIDIN (CHR-BSD)* construct, the 711-bp *CHR* coding region was amplified by PCR using the pRSETb-*mCHERRY* vector as template and primers RBF634 and RBF600. The resulting fragment was digested with *BglII-NotI* and inserted into the pLEXSY-hyg2 vector [22]. In this case, the *HYG* resistance cassette was replaced by the *BSD* ORF, which confers resistance to the antibiotic blasticidin S [23]. A *BSD* resistance cassette was amplified from pXG-*BSD* using the oligonucleotides RBF676 and RBF677 as primers. The amplified 399-bp fragment was digested with *SpeI*-*XbaI* and inserted into pSKII digested with *SpeI*-*XbaI* to generate the pSKII-*BSD* vector. The UTR2 from pLEXSY-hyg2 vector was amplified by PCR using the oligonucleotides RBF772 and RBF773 as primers. The amplified 1339-bp fragment was cut with *NotI-SpeI* and integrated into pSKII-*BSD* vector previously cut with the same restriction enzymes, to generate the pSKII-*BSD*-UTR2 vector. The 1738-bp UTR2-*BSD* fragment was then generated after digestion with *NotI-SpeI* and inserted into the pLEXSY-*CHR*, which was previously cut with the same enzymes, yielding pLEXSY-*CHR*-*BSD*.

Parasites expressing *CHR-BSD* and *CTN-HYG* reporters linked to their corresponding antibiotic-resistance cassettes were obtained after the electroporation of *L. infantum* promastigotes with the large *SwaI* targeting fragment from pLEXSY-*CHR-BSD* and pLEXSY-*CTN-HYG* vectors and plated on semisolid medium [24]. Transgenic *L. infantum* promastigotes derived from the integration of both reporters into the leishmanial 18S rRNA locus were selected on semisolid medium containing 200 µg/ml of hygromycin B or blasticidin S, respectively and confirmed by PCR analysis. In addition, a strong fluorescent signal from both mCherry and Citrine was detected by flow cytometry (Cyan ADP, Dako) and fluorescence microscopy (Nikon Eclipse TE2000E). Parental clones *CHR+ L.infantum* and *CTN+ L.infantum* (hereafter referred to as *CHR+* and *CTN+*) were used to infect mice in order to recover the lost infectivity after cloning and plating.

### Promastigote co-cultures and Balb/c co-infections as platform to generate hybrids

In order to assess the capacity of *L. infantum* to create hybrid progeny *in vitro*, both parental lines were co-cultured. Promastigotes transformed from lesion amastigotes (spleen), were mixed at an equal ratio (1∶1) in M199 medium. Double antibiotic selection was started once the culture reached the stationary growth phase. Hygromycin B and blasticidin S were added on days 7, 11 and 15 after mixing the parental lines. All parasites were dead by this time. The experiment was repeated three independently times including three replicates. In parallel, parental lines that had recovered their infective capacity by passing independently through mice were used to co-infect BALB/c mice. A mix (1∶1) of stationary cultures (2×10^7^) was intraperitoneally injected into female mice (8-weeks old) in a single experiment using 5 animals. Five weeks post-infection, mice were sacrificed and spleens and livers were dissected and processed through a wire mesh and cultured in M199 plus 10% FBS at 26°C. Five days later, promastigotes were seen on cultures. Double drug selection was applied at days 5, 9 and 13. All parasites were dead by this time.

### Experimental sand fly infections

A local colony of *P. perniciosus* sand flies (Madrid) was maintained at 26°C, with a 17-h light/7-h dark photoperiod and 95–100% of relative humidity at the Medical Entomology Unit of the Instituto de Salud Carlos III, Madrid (Spain). This colony was established in 1987 from sand flies captured at a *Leishmania* endemic area of Madrid. Two different protocols were carried out. In the first approach, 3-day old female sand flies were infected by feeding through a chick skin membrane on heat-inactivated rabbit blood containing a mixture (1+1) of logarithmic phase promastigotes of *CHR+* and *CTN+* at a cell density of 2×10^7^ promastigotes/ml. Eight days post-feeding sand flies were dissected in sterile saline and gut contents were transferred directly to 96-well plates containing 0.2 ml media without antibiotics. Eleven days post-blood meal, a new session of dissections among surviving sand flies was done. In the second protocol 5-day old female sand flies were fed with a mixture (1+1) of 1×10^7^ logarithmic phase promastigotes of *CHR+* and *CTN+* promastigotes/ml. Sand fly midguts were dissected 6 days after feeding and the number of viable parasites was determined by counting in a hemocytometer chamber. Hybrid clones were selected by growing the recovered parasite mixture in 24-well plates containing 1 ml of M199 medium supplemented with 100 µg/ml of both hygromycin B and blasticidin S. Fresh M199 medium and antibiotics were replaced every 5 days. PCR was performed to confirm the integration of the *CHR-BSD* and *CTN-HYG* cassettes in the 18S rRNA locus and Southern blot analysis was used to confirm genetic exchange between both parental strains within the sand fly gut. Confocal microscopy was also conducted to confirm the hybrid phenotype.

### *In vitro* infections

THP-1, the human acute leukemia monocyte cell line (ATCC TIB-202), was cultivated in RPMI medium supplemented with 10% heat-inactivated FBS and 1% streptomycin/penicillin at 37°C and 5% CO_2_. The cultures were diluted every 3 or 4 days to maintain the cell density between 10^5^ cells/ml and 8×10^5^ cells/ml. THP-1 cells at 5×10^5^ cells/ml were differentiated with 50 ng/ml of phorbol 12-myristate 13-acetate (PMA) for 48 hours at 37°C and 5% CO_2_. Stationary promastigotes of each parental and hybrid strains (5–6 day cultures) freshly transformed from lesion amastigotes were added to differentiated-macrophages at 1∶10 ratio for 24 h at 37°C. Then, the parasites that have not been internalized were removed by washing with phosphate buffered saline (PBS). Intracellular infections were analyzed after 72 h.

### *In vivo* infections and parasite quantification

Strains infectivity was recovered passing through BALB/c mice. Female BALB/c mice (5 animals per group) were injected intraperitoneally with either 10^7^ infective-stage metacyclic promastigotes of *L. infantum* BCN 150 wild type, *CHR+*, *CTN+* or Hybrid1. Stationary cells freshly transformed from lesion amastigotes were used for the negative selection of metacyclic parasites with peanut agglutinin [25]. After 5 weeks of infection, these animals were euthanized and their spleens and livers were dissected and weighed. The total number of living parasites in the organs was calculated from single-cell suspensions that were obtained by homogenization of the tissue through a wire mesh. Briefly, liver and spleen homogenates (100 mg/ml) were serially diluted in complete Schneider's medium and distributed in 96-well plates at 26°C. After 10 days, each well was examined and categorized as positive or negative according to the presence of viable promastigotes. The number of parasites was calculated as follows: Limit Dilution Assay Units (LDAU)  =  (geometric mean of titer from quadruplicate cultures)×(reciprocal fraction of the homogenized organ added to the first well). The titer was the reciprocal of the last dilution in which parasites were observed [26].

### DNA content analysis

Total DNA content was determined by flow cytometry following staining of 10^6^ permeabilized RNase-treated cells with propidium iodide. *L. infantum* promastigotes (5×10^6^) were washed with PBS and fixed with 70% methanol–30% PBS for 30 min at 4°C. Parasites were pelleted, washed twice with PBS, resuspended in 1 ml of PBS containing 20 µg/ml propidium iodide and 200 µg/ml RNase A, and incubated at 25°C for 1 h. Data were acquired on a FACS flow cytometer (Becton Dickinson), counting at least 10,000 cells per sample, and analyzed using CellQuest 3.1 (BD Bioscience) software.

### Arginase activity and nitric oxide content

The alternative activation of macrophages was assessed by means of determining the arginase activity using the rate of urea yielded from L-arginine in the presence of the chromophore 1-phenyl-1,2-propanedione-2-oxime (ISPF) at 540 nm [27]. The amount of nitric oxide (NO) from infected spleens was indirectly measured using a colorimetric assay based on the Griess reaction [28].

## Results

### Hybrids are generated on the sand fly *P. perniciosus*

The parental clones were tested for their ability to generate doubly drug-resistant parasites during co-infections on the sand fly. In the first protocol a total of 289 sand flies were fed, and after 8 days, 66 of them were dissected and 21 midguts were positive for promastigotes (21/66) giving a transmission index of 32%. Eleven days post-blood-feeding, 54 new dissections among surviving sand flies were done resulting in 20 positive flies. However, due to fungal contamination, only 15 samples had a “clean gut” (15/54) giving a transmission index of 27%. A mix of green and red parasites was detected after sand fly dissections. No promastigotes survived after double drug selection.

In the second protocol 373 sand flies were fed and six days later, 35 were dissected, and 23 guts were positive for promastigotes (23/35) giving a transmission index of 65%. The number of promastigotes per midgut was between 2000 and 5000. Using this protocol only one gut was lost due to fungal contamination. Microscopic analysis showed a mixture of green and red parasite and an intermediate orange color promastigotes after dissection of the sand flies (data not shown). Two populations (isolated from different flies) were resistant to both drugs giving a recombination rate per doubly infected sand fly of 8%. For each doubly drug-resistant population, only a single clone was selected for further analysis.

### Hybrid clones displayed a hybrid genotype and a yellow phenotype

Once double antibiotic selection was done, clonal promastigotes were recovered by plating them on semi-solid media containing both drugs. Only one clone from each hybrid was selected. Therefore, all the molecular work was conducted with two hybrid clones.

**Figure 1:**
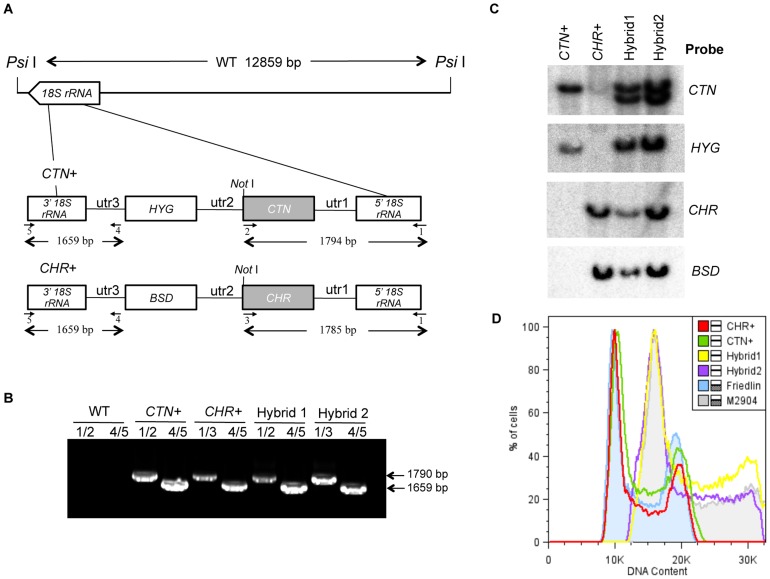
Generation of parental clones and molecular characterization of the hybrid progeny. A) Schematic view of one copy of the 18S rRNA loci on WT and planned integration of the stably-transfected *L. infantum*. B) PCR amplification of the genes encoding fluorescence and antibiotic resistance reporters using the following primers: 1 (RBF630), 2 (RBF614), 3 (RBF600), 4 (RBF644), 5 (RBF645). C) Autorradiography showing Southern blot analysis using four different probes (*CTN*, *HYG*, *CHR* and *BSD* ORFs), in parental (*CTN+*, *CHR+*) and two hybrids. DNA was digested with *PsiI* combined with *NotI*. The DNA amount loaded in lane 3 (Hybrid1) is lesser than in other lanes. D) DNA contents of parental and Hybrid 1 and 2 clones. *L. major* Friedlin FV1 and *L. braziliensis* M2904 were introduced as representative (diploid and triploid) strains in the analysis.

PCR analysis with specific primers for parental markers (*HYG*, *BSD*, *CTN* and *CHR*) (Fig. 1A) showed that all doubly drug-resistant clones contained both genes encoding the antibiotic resistance proteins and both reporter genes. The proper location of the marker genes within the 18S rRNA locus was confirmed for all the clones (Fig. 1B).

Southern blot analysis was performed for four different genes (*CTN* and *CHR* as reporter genes, and *HYG* and *BSD* as antibiotic cassettes). Figure 1C showed that all doubly drug-resistant clones tested contained the *HYG* and *BSD* drug resistance genes as well as the *CTN* and *CHR* reporter genes. However, two hybridization bands corresponding to *CTN* were detected. The smaller size of one of them could be the result of chromosomal rearrangement. This might suggest that both hybrid clones have inherited two chromosome copies from the *CTN+* and one chromosome copy from *CHR+*.

In order to confirm the hypothesis of a triploid hybrid strain, total DNA content of the progeny clones was measured and compared to that of the parental strains by flow cytometry. *L. major* Friedlin FV1 and *L. braziliensis* M2904 were introduced in the analysis as representative “diploid” and “triploid” strains respectively [5]. While parental strains showed 2n DNA contents, hybrid progeny showed 3n DNA contents, their pattern being similar to *L. braziliensis* M2904 (Fig. 1D). These results suggest that the hybrid had inherited two genomic complements from the *CTN-HYG* and one from *CHR-BSD* parents.

**Figure 2:**
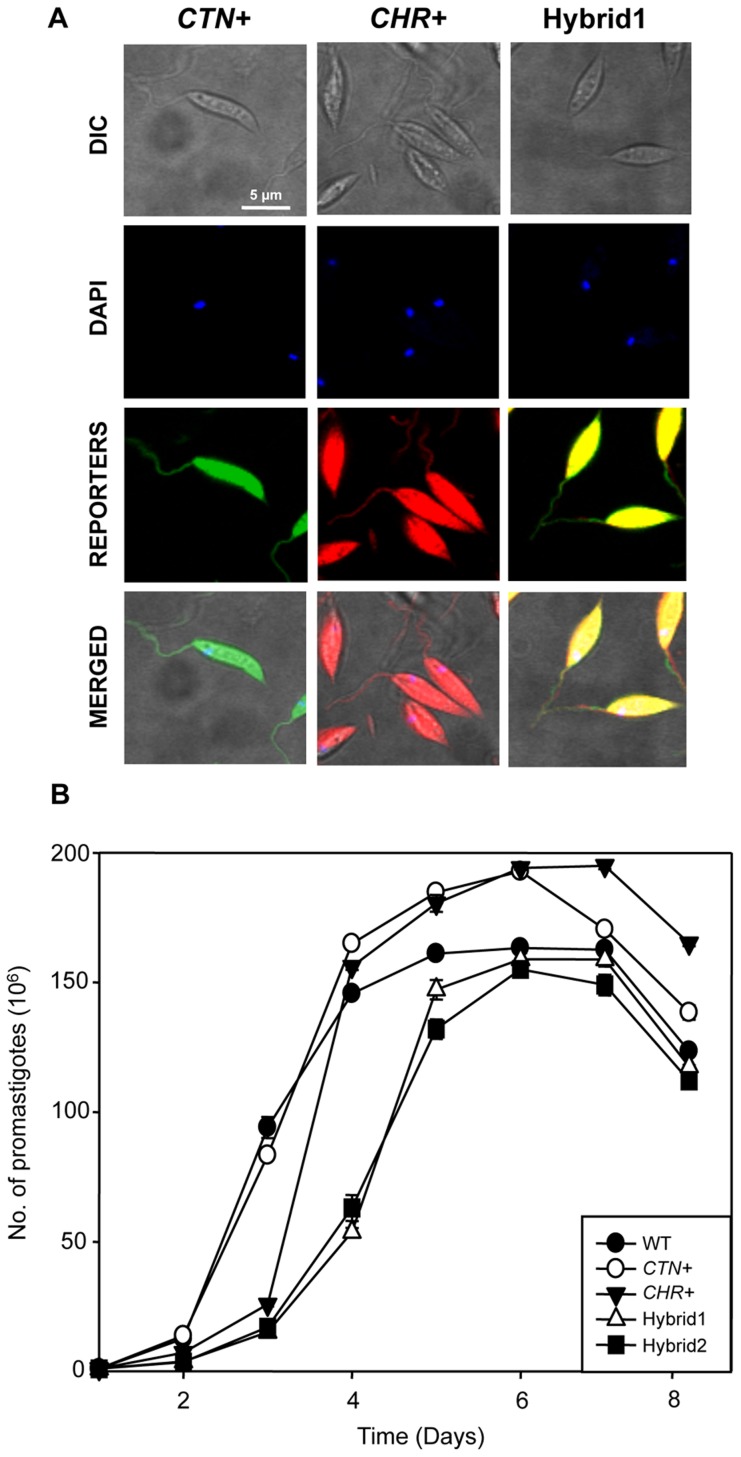
Confocal microscopy analysis of parental and hybrid clones and comparative growth rate analysis. A) *L. infantum* promastigotes were grown in M199 at 26°C in the presence of 100 µg/ml hygromycin B and blasticidin S. Microscopy images were acquired with a Nikon Eclipse TE2000E confocal microscope. B) Growth rate of parental and hybrid promastigotes in the presence of the corresponding selection antibiotics. Cell density was measured by Coulter. This figure only shows Hybrid1 (Hybrid2 grows similarly, data not shown). Experiments were carried out by triplicate and error bars represent standard deviations.

The yellow fluorescent phenotype was detected from doubly drug-resistant hybrids (both hybrids were yellow, only Hybrid1 is shown) (Fig. 2A). The growth rate of both hybrids as promastigotes in M199 with selection antibiotics (hygromycin B and blasticidin S) was slower than that observed in parental lines in the presence of drugs (Fig. 2B). This effect disappeared when drugs were removed (data not shown).

**Figure 3:**
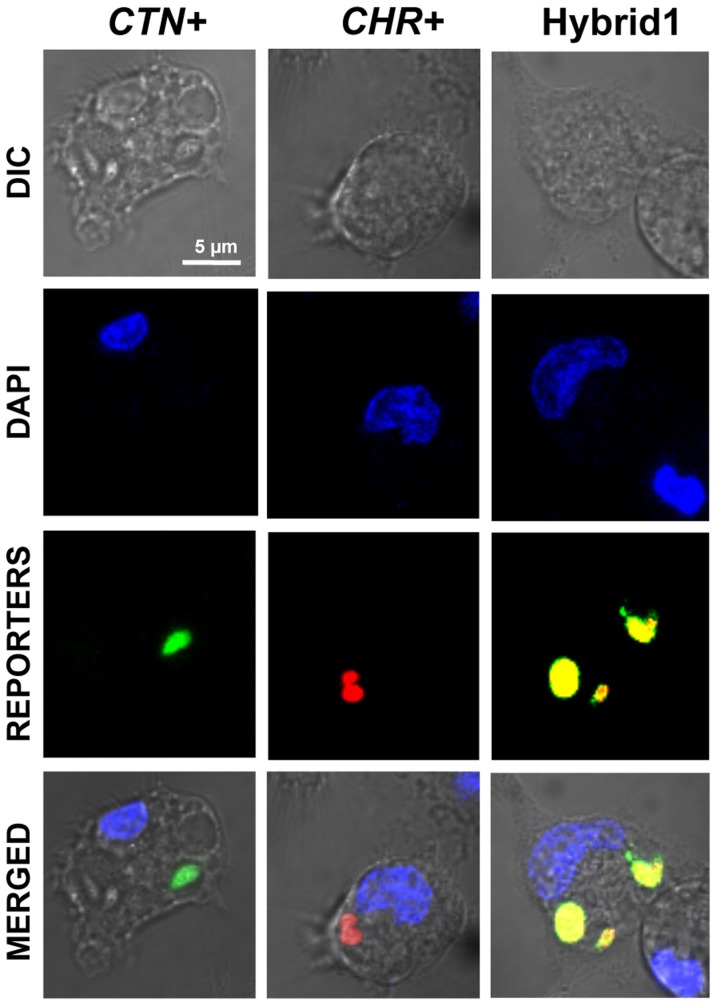
Hybrid parasites are stable and retain yellow fluorescence throughout the whole parasite life cycle. Metacyclic promastigotes (parental and Hybrid1 clones) transformed from amastigotes were co-cultured with differentiated human THP-1 macrophages (ratio 10∶1) at 37°C and 5% CO_2_. After 24 h the wells were washed and the infected macrophages were maintained under similar conditions for further 48 h. Microscopy images were acquired with a Nikon Eclipse TE2000E confocal microscope.

Since the genotype, determined by PCR and Southern blot, was similar for both hybrids, mice infection experiments were performed only with one of the hybrids (hereafter referred to as Hybrid1). To confirm that hybrid formation was stable throughout the complete parasite life cycle, BALB/c mice were injected intraperitoneally with 10^7^ stationary-phase Hybrid1 promastigotes. Five weeks post-infection, animals were sacrificed and parasites were recovered from infected organs (liver and spleen). Freshly transformed promastigotes were again analyzed by confocal microscopy and the stability of the progeny was confirmed through the analysis of total DNA contents (data not shown). Freshly recovered parasites were used to infect differentiated THP-1 macrophages, where the yellow phenotype was conserved (Fig. 3), thus confirming that the hybrid was stable throughout the whole parasite life cycle.

### The hybrid differs in virulence traits in BALB/c mice and macrophage infection

**Figure 4:**
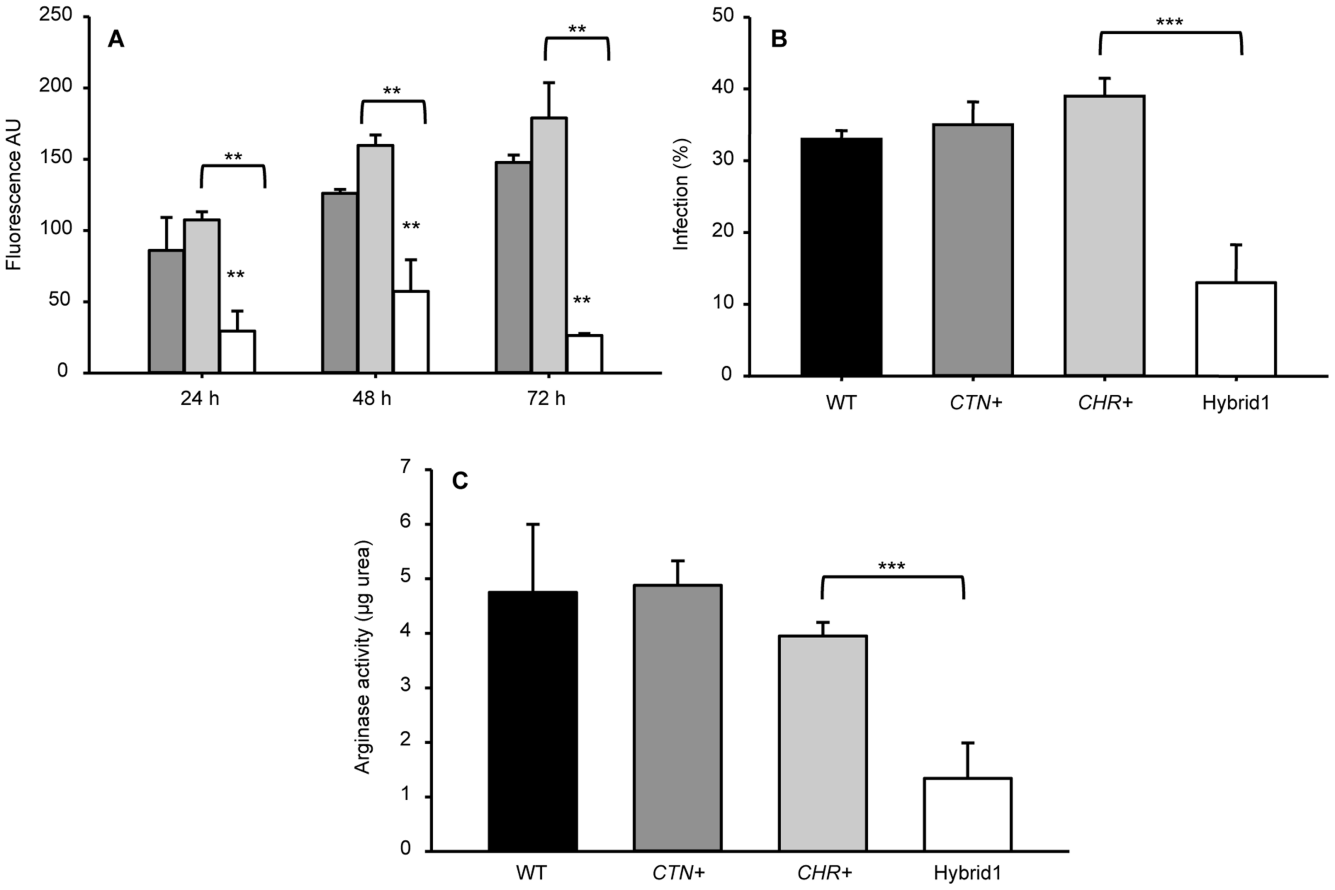
Low infection rate and proliferation impaired in Hybrid1 clone. Differentiated THP-1 macrophages were infected with WT [black]), parental strains (*CTN+* [dark gray], *CHR*+ [light gray] or Hybrid1 [white] and free parasites were washed away after 24 h. A) Time course of the fluorescence emitted by the parental clones and the hybrid progeny. B) Infection rate determined by Giemsa staining of the macrophages infected with the parental and hybrid lineages after 72 h post-infection. C) Total arginase activity was determined as described in the Materials and Methods section. Data are presented as the mean ± standard deviation and are representative of two different experiments with similar results; **, p<0.05; ***, p<0.001.

The ability of parental and hybrid strains to survive and replicate in differentiated THP-1 macrophages was assessed. The time-course of fluorescence emitted by infected macrophages showed significant differences between parental and hybrid clones. The former exhibited higher infection and replication rates, which is represented by an increase in the fluorescent emission along the time-course. The latter showed lower infection rates and its proliferation ability was impaired (Fig. 4A). These results were also confirmed by the percentage of infected macrophages using Giemsa staining. A wild-type (WT) strain was also included only for comparative purposes (Fig. 4B). The arginase activity was significantly reduced in the Hybrid1 in comparison with to parental strains (Fig. 4C).

**Figure 5:**
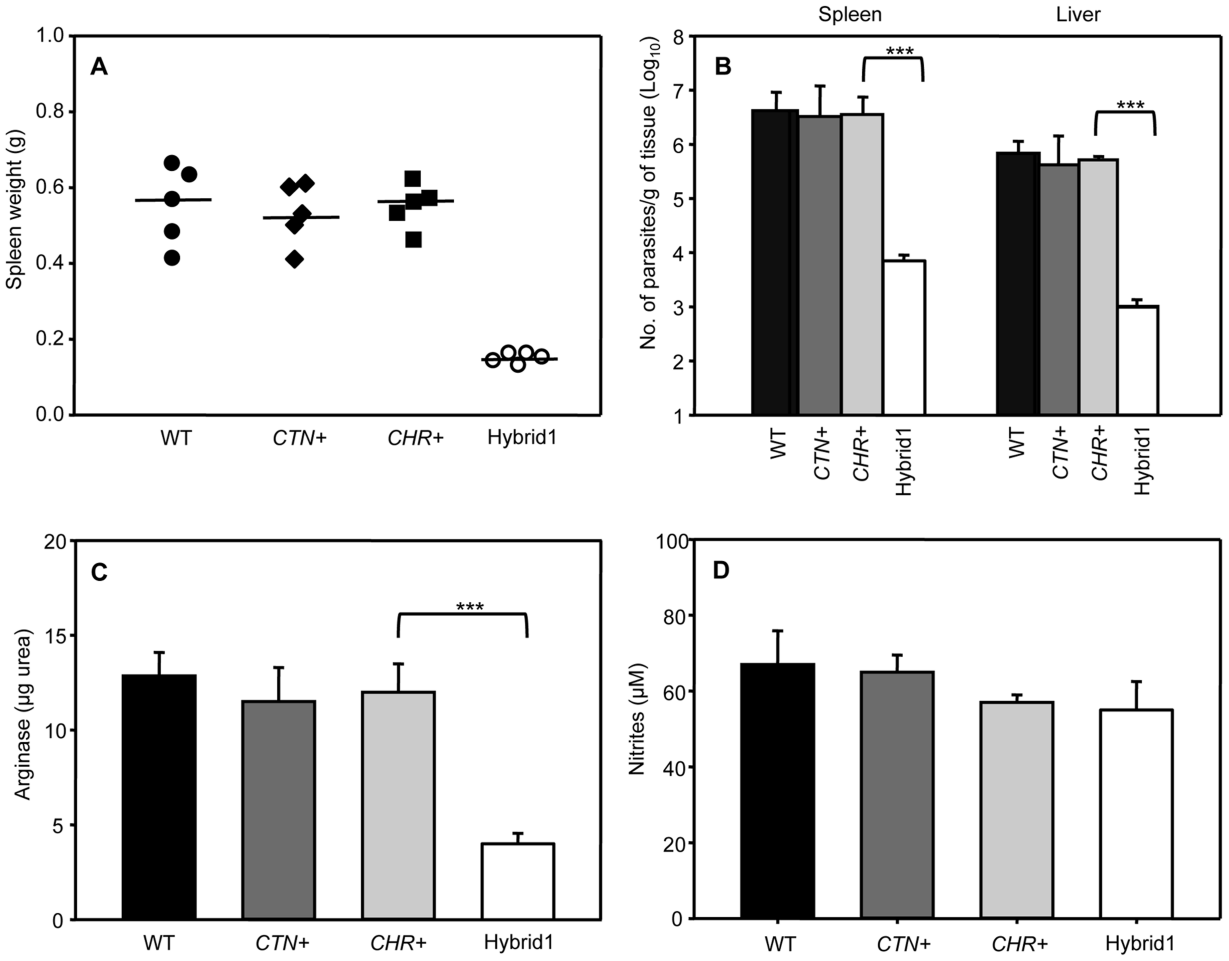
Reduced virulence of Hybrid1 clone. BALB/c mice were infected intraperitoneally with 2×10^6^ (stationary phase promastigotes) WT, parental (*CTN+*, *CHR+*) or Hybrid1 clones. A) Spleen weight of infected animals five weeks post-infection. B) Parasite load per gram of tissue (spleen and liver). C) Total arginase activity from spleen. D) Nitric oxide levels from cultured splenocytes. Data are presented as means ± standard deviation and are representative of three independent experiments with similar results; ***, p<0.001.

Progression of infection in BALB/c mice by parental and hybrid progeny clones was analyzed 5 weeks post-infection. Spleens from infected mice were 4–7 fold bigger than those dissected from un-infected mice (ca. 0.1 g, data not shown), with the exception of mice infected with Hybrid1 clone, whose spleen weight values were around 0.2 g (Fig. 5A).

Total parasite load of spleens and livers was measured using the LDA method. Parasite load per gram of tissue was 10^6^–10^7^ in liver and spleen, respectively in all infected mice with the exception of those mice infected with the Hybrid1 clone. In this case, parasite load was 3 to 4 log lower (liver and spleen, respectively) (p<0.001) than with the WT or parental strains (Fig. 5B). Freshly recovered parasites were grown to stationary phase and peanut agglutinin was used to agglutinate preferentially procyclic parasites. Negative selection of metacyclics showed no differences between different cell lines (parental and Hybrid1) (data not shown).

To gain further insight into the different virulence traits among the parental and Hybrid1 clones, we assessed arginase activity and nitric oxide (NO) production in splenocytes isolated from BALB/c mice infected with parental and Hybrid1 clones. Arginase activity remained similar in WT and parental strains. However, a significant decrease was observed in mice infected with Hybrid1 (Fig. 5C). When supernatants from these cultures were analyzed for nitrite formation by means of the Griess assay, similar values were obtained in all cases (Fig. 5D). Despite the differences in arginase activity induced by parental and Hybrid1 clones, there was no correlation with NO production, which suggests its insignificant role in disease progression. These results indicate that the reduced pathology found in mice infected with Hybrid1 was not the result of increased parasite clearance due to a competitive advantage to metabolize L-arginine via the inducible NO synthetase pathway, but to the impaired virulence of the hybrid line.

## Discussion

In this study we have isolated for the first time in *L. infantum* doubly drug-resistant intraclonal yellow hybrids, which confirm the capacity of *Leishmania* to experience mating within the sand fly vector. Using a system that co-express two reporter genes, two yellow hybrid clones were isolated in a straight-forward manner, extending the genetic exchange not only to cutaneous but also to visceral strains. Additionally, yellow hybrids were generated with individuals of the same *L. infantum* strain, which suggests that *Leishmania* is capable of performing recombination between the same genotype. The percentage of yellow population recovered from total dissected midguts, (2/59) was 3.4%. However, 8–13% recovery was reached in co-infections with *L. major* species [10], [11] and up to 20% (25/121) in experiments with *L. donovani*, [12] although finally none of them were able to survive. Although the low number of hybrid progeny recovered in the present study might suggest that the occurrence of mating in visceral strains is lower than in cutaneous ones, the estimation of meiosis events of *L. infantum* isolates from a focus of cutaneous leishmaniasis in Turkey, gave similar values to those found in experimental trials [6]. The most plausible explanation to the low number of hybrids recovered maybe that intraclonal mating is more unlikely than inter-clonal mating. These results are in concordance with those obtained with the agent responsible for African trypanosomiasis after intraclonal crossing [29].

In the first approach carried out with flies dissected only at late time points (8–11 days), the hybrid recovery was null. Surprisingly, although a time-course was not the aim of this work, shorter co-infection experiments rendered two yellow hybrids by day 6. Previous studies with *L. donovani* co-infecting *P. perniciosus* did not yield viable hybrids, although yellow progeny was described as soon as 3 days after co-infection [12]. Similarly, early hybrid formation (6–8 days post-infection) was pointed by Inbar *et al.* using *L. major* strains [11]. The frequency of genetic exchange in the co-infected sand fly experiments was 2–5×10^−4^. Data were roughly comparable in all the previous studies ranging from <10^−4^ to 2.5×10^−5^.

For both hybrids, the inheritance of four parental selectable markers was confirmed by PCR. Further evidence of chromosomal recombination was the presence of two *CTN* copies of different sizes in the hybrid progeny. The smaller size of one of the copies might be explained by chromosomal rearrangements outside the integration locus. However, parental controls showed that the marker loci had not undergone rearrangement during hybrid formation, thus maintaining their proper location within the 18S rRNA locus. Total DNA content analysis showed that both hybrids were triploids. No diploid, tetraploid or intermediate DNA contents were detected, although the presence of a small number of aneuploid chromosomes may not have been detected by the methods employed in this study.

Hybrid1 was used to infect mice. Recovered promastigotes transformed from lesion amastigotes had yellow phenotype, maintained the doubly drug-resistance and the 3n DNA contents, which evidenced that they were stable throughout the whole parasite life cycle. Ploidy analysis by flow cytometry showed that most of the hybrids recovered from *L. major* and *T. brucei* were diploid like the parental lines, but a significant percentage of hybrids with increased DNA contents (3–4n) was also detected [10], [11], [30]–[32]. This effect was also observed when double gene replacement was conducted in order to knock down an essential gene [33]. *Leishmania* genome contains ∼8000 predicted protein-coding genes and is approximately 33 Mb in size [34]. A 1.5–2 increase in DNA content, equivalent to 16.5–33 Mb, can be only reasonably explained by the duplication of chromosomal material either endogenously or through genetic exchange. Triploids have been considered most likely to be formed by models in which one diploid parental strain omits meiotic reduction and fuses with a haploid gamete provided by the other progenitor (i.e. 2n+n) [35]. Mendelian segregation is well established since long in *T. brucei* [36] and more recently meiosis-specific proteins were expressed in the nucleus mostly before cell fusion [37]. In addition, a promastigote-like cell with haploid DNA contents has been described [38], demonstrating that this excavate protist is essentially a sexual organism. Orthologs of the meiosis-specific genes are present in the *L. infantum* genome and therefore, further studies to demonstrate their functionality during infections in sand fly should be conducted to definitely establish the meiotic program in *Leishmania*. In this scenario triploid hybrids can be generated by the previously proposed idea of fusion between haploid and diploid cells.

The viability and virulence of the *L. infantum* hybrid clone are also important results. The increased arginase activity has been associated with the disease status in patients with visceral leishmaniasis. A local depletion in L-arginine impairs the ability of CD4 T cells to proliferate in the lesion and produce IFN-γ [39]. In this study, the decrease in arginase activity expressed by both THP-1 and splenocytes infected with the Hybrid1 clone suggests a “slow virulence phenotype” in disease progression, and this has not been associated with the growth rate. In *Candida albicans*, tetraploid strains have been described to be less virulent than isogenic diploid strains [40]. Adaptation to the mammalian host environment involves stressful conditions, which requires a particular protein expression pattern that might be impaired by ploidy. Co-infections in sand fly generate polyploid strains. However, there are no reports about the latter among clinical isolates. In *Leishmania* the absence of polyploids in clinical isolates can be explained by two mechanisms: loss of virulence in mice (for 4/5 tetraploids [32]) and reduction to diploid stage [11]. Experiments aimed to analyze gene expression in order to determine differences associated to ploidy must clarify this question.

In summary, *L. infantum* retains the capacity of mating within its natural sand fly vector. Mating is a non-obligatory event in the cell cycle of *Leishmania* triggered by the presence of a unique strain in the sand fly. A clear slower virulence has been shown. It could be a positive feature for the parasite, since it allows the transmission from cryptic hosts to vectors, spreading the disease through endemic areas.
