# Correction: First Evidence of Intraclonal Genetic Exchange in Trypanosomatids Using Two *Leishmania infantum* Fluorescent Transgenic Clones

**DOI:** 10.1371/journal.pntd.0004741

**Published:** 2016-05-17

**Authors:** Estefanía Calvo-Álvarez, Raquel Álvarez-Velilla, Maribel Jiménez, Ricardo Molina, Yolanda Pérez-Pertejo, Rafael Balaña-Fouce, Rosa M. Reguera

The following information is missing from the Funding section: Instituto de Salud Carlos III and Fondos Feder (PI12/00104) partially supported this research.
